# Knowledge, attitudes, and preparedness of healthcare providers trained to respond to violence against women: evaluation of the adapted World Health Organization curriculum in two municipalities in Timor-Leste

**DOI:** 10.1186/s12913-025-12627-7

**Published:** 2025-05-29

**Authors:** Cory N. Spencer, Xylia Ingham, Guilhermina de Araujo, Laurensius Amir, Kayli Wild

**Affiliations:** 1 Department of Global Health, University of Washington, Seattle, WA USA; 2 School of Medicine, University of North Carolina, Chapel Hill, United States; 3 HAMNASA (Hamutuk Nasaun Saudavel– Together a Healthy Country), Formerly Health Alliance International, Dili, Timor-Leste; 4 Institute of Human Security and Social Change & Judith Lumley Centre, La Trobe University, Melbourne, Australia; 5 Menzies School of Health Research, Charles Darwin University, Darwin, Australia

**Keywords:** Violence against women, Training, Health system improvement, Healthcare providers, Timor-Leste

## Abstract

**Background:**

Addressing violence against women (VAW) has been identified as a global health priority, and healthcare providers (HCPs) have an important role in supporting survivors of violence. Despite global advocacy for change, VAW has historically received less attention than infectious or chronic diseases, particularly when health system resources are scarce. To help address these challenges, the World Health Organization (WHO) curriculum on responding to VAW in LMICs was adapted as in-service training in Timor-Leste in 2020–2021, with the goal of training HCPs in two municipalities. We evaluated the impact of the curriculum on HCP knowledge, attitudes, and preparedness using a pre- and post- study design.

**Methods:**

HCPs were recruited from all health facilities in Liquica and Ermera and trained over five days. An adapted version of the WHO curriculum questionnaire was administered pre- and post-training. Using the Wilcoxon signed-rank test, we compared HCPs’ pre- and post-training scores in each domain (*n* = 193). Individual and training-level factors associated with improvement were investigated via regression modeling.

**Results:**

Only 4.7% of HCPs had previously received training on responding to VAW. Median total scores for knowledge of the clinical signs of violence (6.67 vs. 8.89), attitudes towards the unacceptability of violence (6.43 vs. 8.57), individual preparedness (5.67 vs. 8.67), and perceived system support (8.33 vs. 10.0) significantly increased post-training. Attitudes were less likely to improve for older participants (Odds Ratio = 0.42, 95% UI = 0.18–0.95).

**Conclusions:**

This evaluation supports existing evidence that in-service training for HCPs on responding to VAW is effective. The model of engaging all health staff – including medical doctors, nurses, midwives, and other positions – has the potential to shift the culture of care within municipal health systems. Ongoing phases of implementation, including regular follow-up support, present the opportunity to collectively address system barriers to providing survivor-centred care and should be further evaluated.

**Supplementary Information:**

The online version contains supplementary material available at 10.1186/s12913-025-12627-7.

## Introduction

Violence against women (VAW) is a global human rights problem and is responsible for a substantial mortality and morbidity for women and children worldwide [1, 2]. In Timor-Leste, national surveys indicate that 38–59% of women have experienced some form of physical and/or sexual violence from their partner in their lifetime, estimates higher than both global and regional averages [3–5]. Data from the national *Nabilan* survey found that, compared to women who had never experienced violence, abused women had two times higher odds of having symptoms of depression and eight times higher odds of having attempted suicide [4]. Among women in Timor-Leste, experience of violence has additionally been associated with an increase in women’s experiences of miscarriage (*abortu*), sexually transmitted infections, having a baby with low birth weight and having had one or more of their children die [6].

Addressing VAW requires a multi-sectoral approach to engage with governments, communities and services to change entrenched gender norms that enable VAW to continue [7, 8]. In addition to justice and advocacy services, there has been increased recognition for strengthening the role of health systems and healthcare providers (HCPs) in responding to VAW [9]. Survivors of violence are more likely to need healthcare, and HCPs are well-positioned to identify and support these individuals by providing supportive, survivor-centered care and connecting women to other services [10]. However, both systems- and individual-level factors can prevent HCPs from identifying violence and providing appropriate care, including HCP’s poor awareness, harmful attitudes, and a lack of facility supports, such as the availability of guidelines, leadership, time and private spaces [11–13]. Training HCPs on how to respond to VAW in their clinical practice has been found to positively impact HCP engagement with survivors of violence and to increase referrals to other social services [14]. Yet, the majority of evidence on in-service training models and effectiveness is available from high-income settings, which may not face the same challenges and barriers as low-resource settings in establishing a health system-wide response [10].

To address these issues, the World Health Organization (WHO) has published clinical and policy guidelines to strengthen health system and HCP readiness to respond to VAW [15]. In 2019, the WHO published an in-service curriculum, “Caring for women subjected to violence: A WHO curriculum for training health-care providers,” to be used in conjunction with national policy guidelines and referral systems [16]. The curriculum is designed to equip HCPs, particularly those in low- and middle-income countries (LMICs), with the foundational knowledge and skills to implement first-line support and safety for survivors of violence within their clinical practice. Curriculum content includes addressing providers’ attitudes towards survivors of violence, learning how to identify women experiencing violence, and providing first-line support through the LIVES approach (Listen, Inquire, Validate, Enhance safety and Support) [16].

In Timor-Leste, the Ministry of Health (MoH) has developed National Guidelines on the health sector response to VAW [17]. In addition, pre-service training adapted from the WHO curriculum has been implemented by University nursing and midwifery programs in the country [18]. While these steps represent important progress, there is evidence that HCPs in Timor-Leste are still ill-equipped to provide comprehensive services to survivors of violence [13, 18, 19]. Timor-Leste’s public healthcare system was established in 2002 after Timor-Leste gained independence from Indonesia. The tiered health system structure includes one national hospital in the country’s capital, Dili, five regional hospitals, and a network of community health centers (CHCs) and health posts (HPs) across the 13 municipalities within the country. Healthcare services for women are predominately provided by MoH-employed nurses and midwives with support from generalist doctors in the lower-level facilities (CHCs and HPs), and complex medical cases are typically referred to the national hospital in Dili. Women’s access to quality health services remains a challenge in Timor-Leste’s health system [20]. In addition to challenges such as healthcare worker shortages, lack of supplies and lack of access in rural areas, qualitative studies have found a low level of confidence in the skill of providers, which can prevent women from seeking care [20]. Previous research has also shown that many providers lack understanding of supportive approaches for women seeking care for violence and requested more training and support [13, 21]. Additionally, many social services, including VAW services, are concentrated in district centers and Dili, limiting women’s options for seeking help in rural areas.

To help address these service and care gaps, international non-government organization (NGO) Health Alliance International (HAI)^1^, partnered with the MoH, National Institute for Health (INS), Ministry of Social Solidarity and Inclusion (MSSI), and La Trobe University to deliver the *Harmonia Activity*, a USAID-funded, three-year collaborative project that aims to address VAW in Liquica and Ermera municipalities. A key component of the *Harmonia Activity* is the ‘Responding to Gender-based Violence (GBV)’ training for HCPs, based on the WHO curriculum and pre-service adaptation [16, 18]. The ‘Responding to GBV’ curriculum included an initial five days of intensive training, to be followed by monthly ‘learning lab’ sessions to support implementation at all levels of the municipal health system over 14 months. The aim of this evaluation was to assess the outcomes of the five-day intensive phase of the training, including HCP knowledge, attitudes, and preparedness to respond to VAW in their clinical practice. Future work will assess outcomes at the conclusion of 14 months of follow-up learning labs.

## Methods

The project was granted technical and ethical approval by the INS Human Research Ethics Committee in Timor-Leste (ID: 417). The evaluation was reviewed and granted a non-research determination by the University of Washington Institutional Review Board (ID: STUDY00013460).

### Intervention municipalities

Liquica and Ermera were selected as intervention municipalities due to the particularly high prevalence of VAW, with estimates in Liquica and Ermera reaching 33% and 47% higher than the national average (Table 1) [3]. Liquica and Ermera are small municipalities that neighbor the country’s capital, and further characteristics of each municipality are presented in Table 1. The highest tier of health service in these municipalities is a CHC with inpatient beds in the municipal centre, and primary healthcare services are provided by CHCs and HPs in more rural and remote areas of the municipalities (Table 1). There are few specific GBV referral services in rural areas; however, women can be linked with local NGOs, the police vulnerable persons unit, and government assistance, with further specialized GBV and mental health support available in Dili. The aim of the intervention was to train health providers working in health facilities (CHCs and HPs) in each municipality. HAI/HAMNASA staff collaborated with INS, the entity responsible for health provider training and research in Timor-Leste, and with MoH and municipal health authorities to roll out the training in each of the municipalities. All HCP positions were eligible for inclusion in recognition of the role of all health staff and health system leadership in responding to VAW (i.e., medical doctors, midwives and nurses, health facility managers, pharmacists, lab analysts, and other positions).

**Table 1:** Characteristics of Ermera and Liquica municipalities

	Ermera	Liquica	Timor-Leste
**District**			
Capital of the district	Gleno	Liquica	Dili
Subdistricts	Gleno, Atsabe, Ermera, Hatolia, Letefuo, Railaco	Liquica, Bazartete, Maubara	--
**Population** ^**a**^	125,702	71,927	1,183,643
*Male*	63,557	36,436	601,112
*Female*	62,145	35,491	582,531
**Median Age** ^**a**^	17.9	19.7	19.6
**Household Size** ^**a**^	6.1	6.1	5.8
**Currently employed (%)** ^**b**^			
*Men*	91.1	64.6	71.7
*Women*	42.2	42.8	33.7
**Literacy (%)** ^**b**^			
*Men*	72.2	79.3	78.1
*Women*	53.9	72.1	74.6
**Education (median years of school completed)** ^**b**^			
*Men*	5.3	7.0	7.5
*Women*	2.5	6.7	8
**Healthcare facilities and workforce**			
Number of community health centres	7	3	66
Number of health posts	37	27	306
Estimated number of healthcare workers	470	200	3688
**Formal VAW Services Available** ^**b**^	Ministry of Social Solidarity and Inclusion (MSSI), Secretary of State for Equality and Inclusion (SEII), Community police, Health services		PRADET/*Fatin Hakmatek* (psychosocial counselling and support, medical forensic exams, shelter), ALFeLa (legal assistance), JSMP (court monitoring), FOKUPERS/Casa Vida (safe houses), RHTO and CODIVA (advocacy organizations)
**Rates of physical or sexual IPV (%)** ^**c**^			
*Ever*	56.0	50.6	38.1
*Past 12 months*	47.3	48.7	34.6
**Maternal and child healthcare (%)** ^**c**^			
*4 + antenatal visits*	58.8	83.2	84.4
*Birthed with a skilled provider*	19.8	44.8	56.7
*Postnatal care within 2 days*	11.5	32.9	34.5
**Unmet need for family planning (%)** ^**c**^	35.7	22.1	25.3
**Vaccination (%)** ^**c, d**^	30.6	52.4	48.7

^a^Data from 2015 Census [22]

^b^Data from Harmonia Gender Equality and Social Inclusion (GESI) Analysis [19]

^c^Data from 2016 Demographic and Health Survey (DHS) [3]

^d^Percentage of children age 12-23 months who have received all basic vaccinations

### Intervention

The Harmonia Activity “Responding to GBV” five-day training curriculum is based on the WHO’s 2019 in-service curriculum on caring for women subjected to violence [16]. The goal of the WHO curriculum is to equip HCPs in LMICs with the knowledge and skills to implement first-line support and safety for survivors of violence within their clinical practice. The WHO curriculum had previously been adapted in Timor-Leste as a University-level subject, successfully piloted with students, and integrated into the national midwifery curriculum [18, 23]. The original adaptation was contextualized through research with midwives and women-survivors of violence [13, 21, 24] and included the development of Tetum language video resources and case studies, and adaption of the LIVES mnemonic to a Tetum language memory aid *HaHu ReLaSAuN*, which means *Begin a good Relationship* (Ha-Know the signs of violence, Hu-Ask about problems, Re-Respond with empathy, La-Don’t blame the victim, S-Confidentiality, Au-Enhance safety, N-Ongoing support). Based on the positive findings from the pre-service adaptation, the curriculum was further adapted for the *Harmonia Activity* to train currently practicing HCPs. The in-service adaptation included the development of additional follow-up ‘learning lab’ modules designed to support health providers and managers, identify gaps in implementation, and create action plans to assist with quality improvement over time. The Harmonia in-service training curriculum includes both an initial intensive training phase conducted over five consecutive days and an additional 14 months of follow-up support through monthly “learning lab” sessions. This analysis reports the findings from the initial five-day training, with further research planned to evaluate outcomes after the 14 months of follow-up support.

The initial intensive training consisted of 14 modules, aligned with the WHO global curriculum (Supplementary Table 1). Facilitators of the training were employees of HAMNASA and INS, and each facilitator underwent a five-day intensive training-of-trainers (ToT) program which was conducted by lead trainers from the National University (UNTL) and INS, who had previous experience teaching the curriculum and conducting ToT for health lecturers. The training was implemented in-person at municipal health facilities with groups of 15–28 health providers. In addition to structured teaching of content (using PowerPoint slides), the curriculum employed an active learning approach, which aimed to build participant skills and knowledge through discussion, role-play exercises, case studies, videos, and guest speakers. Several modules of the training centered on identifying and building familiarity with local and national services to which survivors could be referred and connecting participants to these services through guest speakers and site visits. From July to November 2021, eleven intensive trainings were facilitated at four health facilities in Ermera and three health facilities in Liquica. A total of 302 health providers participated in the training, representing approximately 45% of health staff in Liquica and Ermera. We followed the Template for Intervention Description and Replication (TIDieR) checklist [25] in reporting our intervention characteristics (Appendix 2).

### Data collection

The pre- and post-training survey was adapted from the WHO evaluation tool, with addition of the Toronto Empathy Questionnaire (TEQ) [16, 26]. Survey questions included basic demographic and work role information and several multi-part questions related to training content and constructs the training intends to change. Participants received pre-training surveys before the first module of training and post-training surveys immediately after the final module. Participants will complete surveys again after 14 months of follow-up ‘learning lab’ sessions. Training staff explained project aims prior to data collection and informed consent was obtained from participants. Surveys were completed by participants on paper forms and data was entered into RedCap by HAI/HAMNASA staff. Data entry was checked for accuracy through a 10% random sample of questionnaires. Each participant was assigned a unique identifier used to match their surveys from the pre- and post-training. A total of 218 participants completed pre-training questionnaires. It was not possible to individually match pre- and post-training survey data for 36 records (25 unmatched pre-training records and 11 unmatched post-training records), resulting in a final analysis sample of 193 participants. Unmatched records were the result of a combination of factors, including attrition from pre- to post-training, failure to collect pre-training data from certain participants, and data collection errors which resulted in the inability to match unique identifiers for certain participants from pre- to post-training.

### Data analysis

#### Pre- and post-training scores by topic area

True/false and yes/no questions were scored 1 for correct answers and 0 for incorrect answers or ‘I don’t know.’ Questions asking about level of participant agreement, acceptability rating, level of preparation, and frequency of actions or feelings were scored on Likert scales from 0 to 3 or 0–4. We present and interpret constructs and topic areas in accordance with the grouping provided in the WHO curriculum questionnaire, which includes scales adapted from the Physician Readiness to Manage Intimate Partner Violence Survey Tool (PREMIS), the Domestic Violence Healthcare Provider Survey Scales (DVHPSS), and the Demographic and Health Surveys (DHS) attitudes towards VAW questions. Additionally, the TEQ is designed to measure empathy and was specifically added to the evaluation tool for this implementation. Many of the scales included in the WHO evaluation tool and the TEQ have not yet been validated in Timor-Leste; therefore, we calculated Cronbach’s alpha for each measured domain using baseline questionnaire data and analyzed only those domains for which Cronbach’s alpha was acceptable (above 0.6; Table 2), indicating content validity. Due to low internal reliability, we did not analyze scales related to appropriate ways to ask about violence, helpful responses to support a woman, attitudes towards gender roles, and empathy; however, we present item-level pre- and post-training mean scores in the appendix (Supplementary Table 2). Specific survey items used to measure each domain included in the main analysis are additionally presented in Supplementary Table 3.

**Table 2:** Related constructs, number of survey items and range of possible points for evaluation topic areas

Construct	Topic area	No. items	Cronbach’s Alpha	Range of possible points
Knowledge	*Clinical signs of violence*	9	0.70	0–9
Attitudes	*Unacceptability of violence*	7	0.85	0–14
	*Attitudes towards asking about violence*	6	0.63	0–24
Perceived Preparedness	*Individual preparedness*	10	0.88	0–30
	*Perceived system support*	6	0.74	0–6

Topic-specific composite scores were derived by summing the scores of each survey item included in the topic area. The number of survey items and range of possible responses differed across domains, so we rescaled composite scores to reflect the possible points proportional to 10 (i.e. all composite scores for each domain range from 0 to 10). Descriptive statistics (median, inter-quartile range) of proportional scores were prepared by evaluation time point (pre/post) and domain. Pre- and post-training data were matched at the individual level using participant’s unique identifiers. Wilcoxon signed rank tests were employed to test for significant differences (α = 0.01) in median scores pre- and post- training, as visual examination of histograms and Shapiro-Wilk tests suggested measures were non-normally distributed. The Holm-Bonferroni correction was applied to p-values to account for multiple comparisons.

#### Participant and training characteristics associated with improvement

To investigate factors potentially associated with average score improvement, we used mixed effects, multivariate logistic regression models with gender, age, position type, municipality, month of training, and whether the participant had ever previously had VAW training as explanatory variables. Month of training was considered as a covariate to account for potential increases in facilitator confidence and competency in delivering trainings as they gained more practice over time. While trainer effects are possible, we were unable to examine these effects by adjusting for trainer identity because several different trainers were responsible for facilitating each of the 14 modules over the five-day intensive period, and each module covered content related to various constructs.

The dependent variable was operationalized as a binary indicator of score improvement pre- to post-training ([post-training score – pre-training score] > 0). For models with more than one topic area included in the construct (i.e., attitudes and preparedness), each model also included a random intercept for each unique participant (regression equation provided in Appendix 1). We ran separate models for each construct, allowing for the assessment of significant covariate effects independently by construct. To account for ceiling effects, observations from participants with pre-training, domain-specific proportional scores of greater than 90% were excluded from datasets used in regression analysis (knowledge of clinical signs of violence, *n* = 27; attitudes towards unacceptability of violence, *n* = 36; attitudes towards asking about violence, *n* = 6; individual preparedness, *n* = 16; perceived system support, *n* = 66). Information on key demographic covariates were missing from 26 participants, who were excluded from all regression analyses. All analyses were performed in R version 3.6.1 [27].

## Results

### Characteristics of the sample

Pre-training data was collected for a total of 218 participants (72.2% of all participants trained), and individually matched pre- and post-training data were available from a total of 193 participants (Table 3). The sample was 61.7% female, and a majority of the sample was 25–34 years old (54.9%). The most common position of training participants across municipalities was medical doctor (31.1%), closely followed by nurse (28.5%) and midwife (24.4%). Other positions included administrators, lab analysts, pharmacists, nursing assistants, and program managers. Very few participants (4.7%) reported having received any previous VAW training at baseline (Table 3).

**Table 3:** Participant demographics and work history by municipality

	Ermera (*N* = 103)	Liquica (*N* = 90)	Overall (*N* = 193)
**Sex**			
Female	60 (58.3%)	59 (65.6%)	119 (61.7%)
Male	37 (35.9%)	28 (31.1%)	65 (33.7%)
Missing	6 (5.8%)	3 (3.3%)	9 (4.7%)
**Age**			
Less than 25 years old	4 (3.9%)	4 (4.4%)	8 (4.1%)
25–34 years old	63 (61.2%)	43 (47.8%)	106 (54.9%)
35–44 years old	17 (16.5%)	26 (28.9%)	43 (22.3%)
45–54 years old	13 (12.6%)	6 (6.7%)	19 (9.8%)
55 years or older	2 (1.9%)	3 (3.3%)	5 (2.6%)
Missing	4 (3.9%)	8 (8.9%)	12 (6.2%)
**Position**			
Community health worker	2 (1.9%)	6 (6.7%)	8 (4.1%)
Medical doctor	33 (32.0%)	27 (30.0%)	60 (31.1%)
Midwife	24 (23.3%)	23 (25.6%)	47 (24.4%)
Nurse	33 (32.0%)	22 (24.4%)	55 (28.5%)
Other	9 (8.7%)	8 (8.9%)	17 (8.8%)
Missing	2 (1.9%)	4 (4.4%)	6 (3.1%)
**Years of practice**			
Mean (SD)	7.36 (6.39)	7.00 (7.27)	7.20 (6.78)
Median [Min, Max]	6.00 [0, 30.0]	6.00 [0, 31.0]	6.00 [0, 31.0]
Missing	31 (30.1%)	32 (35.6%)	63 (32.6%)
**Average weekly patient volume**			
Currently not seeing patients	3 (2.9%)	3 (3.3%)	6 (3.1%)
Less than 20	18 (17.5%)	10 (11.1%)	28 (14.5%)
20–39	16 (15.5%)	16 (17.8%)	32 (16.6%)
40–59	7 (6.8%)	8 (8.9%)	15 (7.8%)
60 or more	20 (19.4%)	20 (22.2%)	40 (20.7%)
Missing	39 (37.9%)	33 (36.7%)	72 (37.3%)
**Previous GBV Training**			
No	97 (94.2%)	87 (96.7%)	184 (95.3%)
Yes	6 (5.8%)	3 (3.3%)	9 (4.7%)

### Changes in knowledge, attitudes, and perceptions of preparedness

At baseline, median scores ranged from 5.67 points (individual preparedness) to 8.33 points (perceived levels of system support; Table 4). Median scores for knowledge of the clinical signs of violence (6.67 vs. 8.89, *p* < 0.001), attitudes towards the unacceptability of violence (6.43 vs. 8.57, *p* = 0.0018), individual preparedness (5.67 vs. 8.67, *p* < 0.001), and perceived system support (8.33 vs. 10.0, *p* < 0.001) significantly increased from pre- to post-training. Slight improvement was found for HCP attitudes towards asking about violence (6.25 vs. 6.67, *p* = 0.038); however, this result was not significant at the α = 0.01 level (Table 4). Across the entire sample, 176 (91.2%) of participants correctly recalled the seven steps of first-line support in the adapted LIVES mnemonic *‘HaHu ReLaSAuN’* at the post-training survey.

**Table 4:** Differences in median scores pre- and post-training by domain

Construct	Domain	*N*	Pre-training	Post-training	z-score	*p*-value*
			Median (IQR)	Median (IQR)		
**Knowledge**	Clinical signs of violence	179	6.67 (5.56–8.89)	8.89 (7.78–10.0)	−8.67	< 0.001
**Attitudes**	Unacceptability of violence	189	6.43 (3.57–8.57)	8.57 (5.00–10.0)	−3.92	0.0018
	Attitudes towards asking about violence	185	6.25 (5.00–7.08)	6.67 (5.63–7.5)	−2.07	0.0382
**Perceived Preparedness**	Individual Preparedness	188	5.67 (4.00–7.33)	8.67 (6.67–10.0)	−9.08	< 0.001
	System support	188	8.33 (5.00–10.0)	10.0 (8.33–10.0)	−7.18	< 0.001

*N *number of participants included in domain-specific analysis

*p-values presented are adjusted using the Holm-Bonferonni correction. Maximum score possible for each domain is 10 points

### Participant and training characteristics associated with improvement

On average across evaluation constructs, probability of improvement did not significantly differ by participant gender, position, municipality, month of training, or previous VAW training (Table 5). For the attitudes construct, participants aged 35 years or older were significantly less likely to improve from pre- to post-training (OR = 0.42, 95% UI = 0.18–0.95) compared to participants younger than 35. Older participants were equally as likely to improve compared to their younger counterparts for knowledge and perceived preparedness constructs (OR = 3.87, 95% UI = 0.44–33.94; OR = 0.66, 95% UI = 0.17–2.57, respectively).

**Table 5:** Multivariate logistic regression estimates

	Knowledge (*n* = 143)			Attitudes (*n* = 166)			Perceived Preparedness (*n* = 162)		
	*Estimate (SD)*	*p*	*Odds Ratio (95% UI)*	*Estimate (SD)*	*p*	*Odds Ratio (95% UI)*	*Estimate (SD)*	*p*	*Odds Ratio (95% UI)*
Female	0.05 (0.54)	0.9291	1.05 (0.36, 3.02)	−0.25 (0.28)	0.3896	0.78 (0.45, 1.37)	0.71 (0.46)	0.123	2.03 (0.83, 4.99)
Age > 35	1.35 (1.11)	0.2224	3.87 (0.44, 33.94)	**−0.88 (0.42)**	**0.038**	**0.42 (0.18**,** 0.95)**	−0.42 (0.69)	0.5464	0.66 (0.17, 2.57)
Nurse	−0.3 (0.57)	0.598	0.74 (0.24, 2.27)	0.12 (0.32)	0.6968	1.13 (0.6, 2.12)	0.17 (0.52)	0.7438	1.19 (0.43, 3.31)
Midwife	−0.39 (0.65)	0.5507	0.68 (0.19, 2.41)	0.11 (0.38)	0.7806	1.11 (0.53, 2.35)	−0.22 (0.61)	0.7197	0.80 (0.24, 2.65)
Other position	1.43 (1.15)	0.2145	4.18 (0.44, 40.09)	0.41 (0.43)	0.926	1.04 (0.45, 2.41)	−0.34 (0.68)	0.6161	0.71 (0.19, 2.69)
Liquica	−0.15 (0.53)	0.7789	0.86 (0.3, 2.45)	−0.33 (0.29)	0.2648	0.72 (0.41, 1.28)	0.10 (0.47)	0.8326	1.10 (0.44, 2.75)
Sep 2021	0.07 (0.59)	0.9108	1.07 (0.33, 3.41)	−0.17 (0.33)	0.6118	0.85 (0.45, 1.61)	0.78 (0.53)	0.1406	2.19 (0.77, 6.19)
Oct 2021	−0.17 (0.72)	0.8072	0.84 (0.21, 3.41)	−0.12 (0.39)	0.7659	0.89 (0.41, 1.91)	1.01 (0.71)	0.1538	2.75 (0.68, 11.04)
Previous VAW training	0.37 (1.14)	0.7467	1.44 (0.15, 13.49)	0.1 (0.58)	0.8584	1.11 (0.35, 3.47)	−0.45 (0.81)	0.5825	0.64 (0.13, 3.14)

For each regression model covariate, the reference categories are listed in parentheses: female (male); age > 35 (age less than 35); Nurse (medical doctor); Midwife (medical doctor); Other position (medical doctor); Liquica municipality (Ermera municipality); September 2021 (July/August 2021); October 2021 (July/August 2021); Previous VAW training (No previous VAW training). Coefficients for domains within a construct are not shown but were included as fixed effects in the model; covariates presented can be interpreted as the average effect of the covariate across all domains included for that construct

*VAW *Violence Against Women, *UI* Uncertainty Interval, *SE* Standard Error, *n* number of participants in each regression analysis

Significant effects (*p* < 0.05) are bolded

## Discussion

This study reports the impact of a five-day training program on health provider knowledge, attitudes, and preparedness to support survivors of violence within two municipalities in Timor-Leste. Participants reported improvements in their ability to identify the clinical signs of violence, more positive attitudes towards the unacceptability of violence, greater preparation for responding to violence, and greater levels of system support. By successfully reaching a variety of health professionals across municipal health services, this training promotes a systems-wide approach to addressing violence from within the health sector.

Evaluation of training interventions to improve HCP responses to violence have not been widely conducted in LMICs, with comparable assessments almost exclusively available from high-income, North American contexts. A recent systematic review of experimental trials of HCP training to improve responses to VAW was composed of 19 studies, 14 of which were conducted in the United States [10]. Among these trials, common measures of impact were changes in HCP knowledge, self-confidence, skills, and attitudes. This study therefore fills a critical gap in evidence for how similar interventions may work to change provider knowledge, attitudes, and preparedness in LMIC settings.

The improvements demonstrated in this intervention are consistent with those that have been found in the few interventions conducted in other LMICs [28–31]. Specifically, a recent implementation of the WHO’s curriculum conducted among healthcare workers in tertiary hospitals in India showed immediate improvements in participants’ knowledge of the clinical signs of violence, attitudes towards the acceptability of violence, individual preparedness, and perceived system support, findings highly aligned with those observed here [30]. However, while attitudes of HCPs towards the unacceptability of violence improved from pre- to post-training, attitudes towards the HCP’s professional role, particularly attitudes toward asking about violence, did not significantly change in both the India and Timor-Leste studies. These outcomes emphasize the difficult nature of measuring and potentially changing provider attitudes toward their role within the broader culture of the health system. Factors that affect HCP’s engagement with women include considering domestic violence as a private issue, the tendency to blame the victim for the abuse, and wider health system barriers [21, 24], all of which underscore the need to continue to address such attitudes in follow-up training sessions to support effective HCP responses.

At the individual level, our multivariate regression showed that age had a significant association with the probability of attitude improvement, with younger participants more likely to demonstrate score improvement from pre- to post-training. This finding is consistent with previous work from the implementation of the WHO curriculum with nursing and midwifery students in Timor-Leste, which showed large improvements in attitudes among a younger cohort [18]. As certain attitudes towards gender and violence may be driven by generational factors, our findings support the need for engagement with providers both pre- and in-service to effect sustainable, systems-level change over time. Importantly, older providers or those who have been working in the health system for longer periods of time may need additional mentoring and follow-up to change their attitudes about VAW and caring for survivors of VAW. Age is linked to authority and leadership roles in many societies, including in Timor-Leste [32]. Thus, addressing VAW requires not only targeted interventions but engagement with community and health systems leaders who can support and cultivate positive organizational changes [13]. Indeed, it is the intent of the curriculum that findings from the initial intensive stage of training be used to customize areas of reinforcement during the 14-months of follow-up in the form of “learning lab” sessions. These sessions provide the opportunity to continue engaging with participants who may not have achieved positive attitude changes during initial training. Those planning adaptations of the WHO curriculum in other LMIC settings may benefit from further focus on curriculum content that supports experiential and transformative learning in addition to sustained follow-up support and encouragement to shift attitudes over time.

While the improvements reported here may be positive precursors to behavior change, the intensive nature of the initial training program prevented the measurement of clinical outcomes and practices. However, similar interventions in other LMIC settings have found improvements in constructs like knowledge and confidence to be concurrent with increases in supportive clinical practices. For example, a four-day VAW training program conducted among public health midwives in Sri Lanka found improvements in self-reported knowledge and self-efficacy along with increases in the discussion and suggestion of solutions with identified women six months post-intervention [28]. Yet, a recent Cochrane review on the effectiveness of training health providers to respond to VAW found more evidence is required to understand the impact of training on HCP’s responses in the long term [10]. The continued engagement of HCPs through follow-up visits to support leadership and implementation will therefore be a critical part of the *Harmonia Activity* in order to work through real-world challenges and improve HCP attitudes and practices over time.

The findings of this evaluation should be interpreted considering certain limitations. First, the training was implemented as part of a capacity-building program by government ministries and an NGO and followed a non-experimental design without a control group. Therefore, it is not possible to fully attribute changes in outcomes to the intervention. However, given the program’s goals and resources, the pre-post study design represents a rigorous evaluation of real-world implementation in an LMIC setting. Second, our instrument, while adapted from the WHO tools and translated/back translated, has not been formally validated in the study context. While we restricted our analyses to domains that demonstrated medium or high internal reliability, we found low internal reliability for many of the scales used in the evaluation tool, including the TEQ, and more work is needed to refine the measurement of challenging constructs like attitudes and empathy in this context. Despite these limitations, there is considerable evidence to support the curriculum’s impact on improved HCP knowledge, attitudes, and preparedness in responding to VAW in their clinical practice. This work also has notable strengths, including a large sample size that captured multiple professions within the primary healthcare system in Timor-Leste and the use of standards and tools adapted from the WHO curriculum, which increases comparability of our findings to those of similar programs in other LMIC settings.

## Conclusions

The  findings from a locally adapted implementation of the WHO in-service curriculum showed the training program improved HCP knowledge, attitudes, and preparedness to support women experiencing violence in two municipalities in Timor-Leste. This evaluation adds to the evidence that in-service training for HCPs to address violence can be effective and that it is possible to reach all primary healthcare facilities with capacity building initiatives in LMIC settings. The low prevalence of previous training found among program participants emphasizes the urgent need for training and ongoing system support in other municipalities. Models of training that are most likely to be successful in changing practice include engaging all providers at the health facility, supporting leadership to listen and identify gaps in service systems, and facilitating continuous improvement of services [14]. HAMNASA is conducting health facility readiness assessments to collectively assess system barriers and identify areas for improvements. These supportive follow-up sessions present the crucial opportunity to reinforce training content in a low-dose fashion, engage providers who may not have been reached by the initial training, and address system barriers such as privacy, time, guidelines, record keeping, referral systems, and community engagement. These more sustained approaches to supporting implementation within health systems should continue to be trialed and assessed for impact on the lives of VAW survivors.

## Supplementary Information

Supplementary Material 1.

Supplementary Material 2.

## Data Availability

The datasets used and/or analysed during the current study are available from the corresponding author on reasonable request.

## Footnotes

1 HAI is no longer operating and transitioned to local NGO HAMNASA in October 2021. The Activity is continuing to be implemented by HAMNASA.
