# Supplementary material for: Knowledge, attitudes, and preparedness of healthcare providers trained to respond to violence against women: evaluation of the adapted World Health Organization curriculum in two municipalities in Timor-Leste
Source: BMC Health Serv Res. 2025 May 29;25:770. doi: 10.1186/s12913-025-12627-7 (PMC12121071; doi:10.1186/s12913-025-12627-7)
Supplement: Supplementary file 1 — Supplementary Material 1. [file 12913_2025_12627_MOESM1_ESM.docx]

Appendix 1: Supplemental information and methods to “Knowledge, attitudes, and preparedness of healthcare providers trained to respond to violence against women: Evaluation of the adapted WHO curriculum in two municipalities in Timor-Leste”

This appendix provides further information and methodological details for “Knowledge, attitudes, and preparedness of healthcare providers trained to respond to violence against women: Evaluation of the adapted WHO curriculum in two municipalities in Timor-Leste”

Contents

[Table S1: Curriculum content and learning objectives by module. 2](#_Toc195478026)

[Table S2. Item-level pre- and post-training mean score differences. 5](#_Toc195478027)

[Table S3. Survey items used in measurement of each topic area and domain analyzed. 12](#_Toc195478028)

[Regression Equation 14](#_Toc195478029)

# Table S1: Curriculum content and learning objectives by module.

| **Module** | **Learning objectives** |
| --- | --- |
| Module 1 – Introduction: Violence against women and children as public health issues | 1.1 Definitions of domestic violence, sexual assault and child abuse |
|  | 1.2 Prevalence of different forms of violence globally and in Timor-Leste |
|  | 1.3 The cycle of violence and who is more vulnerable to being abused |
| Module 2 – Violence and society: Beliefs, attitudes and barriers to getting help | 2.1 Contributors to violence against women and children in Timor-Leste |
|  | 2.2 How to challenge common beliefs and attitudes about domestic violence, sexual assault and child abuse |
|  | 2.3 Obstacles for women getting help |
| Module 3 – The role of health providers: Laws and policies for responding to domestic and sexual violence in Timor- Leste | 3.1 The role and responsibility of healthcare providers within a health system response to violence against women and children |
|  | 3.2 Laws and policies for responding to violence against women and children in Timor-Leste |
| Module 4 – Impact on health and wellbeing: Understanding the consequences of violence and identifying signs of abuse | 4.1 Short and long-term physical and psychological consequences of domestic violence |
|  | 4.2 Physical and behavioral signs of violence in adults |
|  | 4.3 Physical and behavioral signs of violence in children and people with a disability |
| Module 5 – Guiding principles: Woman-centred care, privacy and non-verbal communication | 5.1 Principles of woman-centred care |
|  | 5.2 The importance of privacy for women’s and children’s safety |
|  | 5.3 The impact of non-verbal communication |

| Module 6 – Ask about  problems: Building trust and types of questions to ask | 6.1 Obstacles for healthcare providers asking about violence  6.2 The importance of rapport and trust in facilitating good communication |
| --- | --- |
|  | 6.3 How to raise the subject and know how to ask about suspected abuse |
| Module 7 – First line support  (Part 1): Respond with empathy, do not blame the victim, protect confidentiality | 7.1 How to listen and communicate empathically with clients |
|  | 7.2 Further considerations when assisting children and people with a disability |
|  | 7.3 How to do no harm and avoid re-traumatizing victims of violence |
|  | 7.4 How to protect a client’s confidentiality and explain its limits |
| Module 8 – Enhance safety:  Danger assessment and safety planning | 8.1 How to assess the level of danger for a woman and her children |
|  | 8.2 How to make a safety plan |
|  | 8.3 How to respond to perpetrators of violence |
| Module 9 – Documenting  domestic violence, sexual assault and child abuse | 9.1 How to carefully and confidentially document information about domestic violence, sexual assault and child abuse |
|  | 9.2 When and how to refer for a medical forensic examination |
| Module 10 – Referral services: Formal and informal support for survivors of violence | 10.1 The diverse needs of women and children experiencing violence |
|  | 10.2 Social services and other sources of support in the community |
| Module 11 – Study tour: Visit to referral services | 11.1 How to build a relationship with referral services and strengthen the referral network |
| Module 12 – Ongoing support: Mental health and warm referral | 12.1 How to support mental health and positive coping strategies  12.2 How to link clients to support and provide a warm referral |
| Module 13 – Clinical care for sexual assault: Preventing STIs, HIV and unwanted pregnancy | 13.1 How to identify conditions that require urgent medical care |
|  | 13.2 How to prevent HIV through post-exposure prophylaxis |
|  | 13.3 When and how to provide emergency contraception |
|  | 13.4 Prevention and treatment of sexually transmitted infections (STI) |
| Module 14 – Self-care and creating a supportive health system | 14.1 How to look after the physical and emotional health, and safety of themselves and colleagues |
|  | 14.2 Health system requirements for addressing domestic and sexual violence at different levels of health services |

# Table S2. Item-level pre- and post-training mean score differences.

| **Topic Area** | **Survey Item** | **Pre-training Mean (SD)** | **Post-training Mean (SD)** | **Difference Mean (SD)** | **Non-missing observations** |
| --- | --- | --- | --- | --- | --- |
| **KNOWLEDGE** | | | | | |
| VAW and Healthcare Provider Role^ǂ^ | ***Please indicate whether you think the following statements are TRUE OR FALSE:*** | | | | |
|  | 1. Women who experience violence tend to need health care more often than women who do not experience violence | 0.88 (0.326) | 0.943 (0.232) | 0.0625 (0.377) | 192 |
|  | 1. For cases of rape in Timor-Leste, the perpetrator is most likely to be a stranger. | 0.359 (0.481) | 0.427 (0.496) | 0.0628 (0.538) | 191 |
|  | 1. There are common injury patterns associated with domestic violence. | 0.76 (0.428) | 0.938 (0.242) | 0.177 (0.489) | 192 |
|  | 1. A woman who has been raped needs immediate health-care. | 0.947 (0.224) | 0.99 (0.102) | 0.0421 (0.226) | 190 |
|  | 1. Any disclosure about domestic violence or sexual assault should be treated confidentially. | 0.891 (0.313) | 0.938 (0.243) | 0.0471 (0.387) | 191 |
|  | 1. If a health care provider suspects that a woman has been subjected to violence, it is helpful to ask her about it. | 0.828 (0.378) | 0.948 (0.222) | 0.12 (0.434) | 192 |
|  | 1. If a health-care provider suspects violence but the woman does not open up about it, there is nothing he/she could do to help. | 0.414 (0.494) | 0.667 (0.473) | 0.258 (0.564) | 190 |
|  | 1. Health-care providers should not pressure patients to disclose that they are living in an abusive relationship. | 0.719 (0.451) | 0.751 (0.433) | 0.0312 (0.558) | 192 |
|  | 1. If you suspect the husband is being violent, it is advisable to talk to both the woman and her husband together. | 0.205 (0.405) | 0.839 (0.368) | 0.632 (0.514) | 190 |
|  | 1. The health-care provider should allow the victim to make her own decisions. | 0.853 (0.355) | 0.948 (0.222) | 0.0942 (0.385) | 191 |
|  | 1. The health worker must verify how accurate a woman’s story is by asking the alleged abuser or the woman’s friends and family. | 0.224 (0.418) | 0.472 (0.5) | 0.245 (0.575) | 192 |
|  | 1. Even when it is not clear what the health-care provider can do to help the woman, one thing he/she can do is to listen to her closely. | 0.875 (0.332) | 0.912 (0.284) | 0.0365 (0.413) | 192 |
|  | 1. If the woman starts to cry, the health-care provider should immediately end the interview so she can leave. | 0.806 (0.396) | 0.891 (0.313) | 0.0842 (0.463) | 190 |
|  | 1. It is important not to share or discuss the woman’s information with anyone unless you have talked to her about it first. | 0.823 (0.383) | 0.886 (0.319) | 0.0625 (0.452) | 192 |
|  | 1. It is a health care provider’s legal duty to help the woman subjected to violence to report it to the police. | 0.728 (0.446) | 0.715 (0.453) | -0.0105 (0.597) | 191 |
|  | 1. Women in abusive relationships may have valid reasons for staying together in the relationship. | 0.277 (0.449) | 0.385 (0.488) | 0.118 (0.582) | 187 |
|  | 1. It is important to document the past sexual history of a woman when examining for sexual assault or rape. | 0.762 (0.427) | 0.802 (0.399) | 0.0426 (0.514) | 188 |
|  | 1. Women in abusive relationships may have valid reasons for wanting a divorce. | 0.783 (0.413) | 0.854 (0.354) | 0.0745 (0.489) | 188 |
|  | 1. Children who witness violence against women in their homes or communities are not affected unless they are physically abused themselves. | 0.534 (0.5) | 0.646 (0.48) | 0.106 (0.564) | 188 |
| Clinical signs of violence**^ǂ^** | ***For each of the following, indicate whether or not it is a warning sign that a woman may have been subjected to domestic or sexual violence? (YES/NO)*** | | | | |
|  | a.       Repeated unwanted pregnancy? | 0.745 (0.437) | 0.953 (0.212) | 0.209 (0.444) | 187 |
|  | b.     She has problems with alcohol or drug abuse? | 0.772 (0.42) | 0.911 (0.285) | 0.138 (0.507) | 188 |
|  | c.       Repeated sexually transmitted infections? | 0.759 (0.429) | 0.958 (0.2) | 0.199 (0.45) | 186 |
|  | d.      Chronic unexplained pain or inflammation (e.g. pelvic, headaches)? | 0.489 (0.501) | 0.728 (0.446) | 0.242 (0.559) | 186 |
|  | e.      Frequent injuries? | 0.679 (0.468) | 0.859 (0.349) | 0.178 (0.448) | 185 |
|  | f.        Injuries that do not match the explanation of how they happened? | 0.62 (0.487) | 0.759 (0.429) | 0.145 (0.524) | 186 |
|  | g.       Depression, anxiety or chronic stress? | 0.888 (0.317) | 0.99 (0.102) | 0.102 (0.32) | 186 |
|  | h.      Thoughts, plans or acts of self-harm or attempted suicide? | 0.856 (0.352) | 0.958 (0.2) | 0.107 (0.386) | 187 |
|  | i.        Repeated health consultations with no clear diagnosis? | 0.495 (0.501) | 0.693 (0.463) | 0.209 (0.607) | 187 |
| Appropriate ways to ask about violence**^ǂ^** | ***Read the following statements and indicate whether or not it is an appropriate way to ask about domestic violence? (YES/NO)*** | | | | |
|  | 1. “Are you a victim of domestic violence?” | 0.574 (0.496) | 0.667 (0.473) | 0.0856 (0.673) | 187 |
|  | 1. “Has your husband/boyfriend ever hurt or hit you?” | 0.534 (0.5) | 0.791 (0.408) | 0.257 (0.59) | 191 |
|  | 1. “Does your husband/boyfriend insult you or threaten you?” | 0.549 (0.499) | 0.775 (0.419) | 0.23 (0.605) | 191 |
|  | 1. “Many women experience serious problems in their relationships. Have you had any difficulties in your relationship?” | 0.531 (0.5) | 0.853 (0.355) | 0.321 (0.569) | 190 |
|  | 1. “Are you afraid of anyone in your family?” | 0.615 (0.488) | 0.859 (0.349) | 0.246 (0.558) | 191 |
| Helpful responses**^ǂ^** | ***Read each statement and decide whether or not it is a helpful thing to say to support a woman subjected to domestic violence or sexual assault? (YES/NO)*** | | | | |
|  | 1. “Would you like to tell me more about that?” | 0.786 (0.411) | 0.891 (0.313) | 0.105 (0.445) | 191 |
|  | 1. “How do you feel about that?” | 0.698 (0.46) | 0.869 (0.338) | 0.168 (0.555) | 190 |
|  | 1. “Why did you go there alone, don’t you know it’s dangerous?” | 0.415 (0.494) | 0.562 (0.497) | 0.146 (0.603) | 192 |
|  | 1. “Tell me exactly what he did, you must describe to me all the details.” | 0.335 (0.473) | 0.45 (0.499) | 0.107 (0.584) | 187 |
|  | 1. “You should not feel so sad, you should feel lucky that you survived.” | 0.269 (0.445) | 0.516 (0.501) | 0.245 (0.575) | 192 |
|  | 1. “If it’s so bad, you should just leave him.” | 0.591 (0.493) | 0.797 (0.403) | 0.208 (0.567) | 192 |
|  | 1. “I am worried that the violence may be affecting your health and your children’s health.” | 0.881 (0.325) | 0.969 (0.174) | 0.0885 (0.378) | 192 |
|  | 1. “Trust me, I know that this option will be the best for you.” | 0.328 (0.471) | 0.453 (0.499) | 0.132 (0.581) | 189 |
|  | 1. “I can help you make a plan for you and your children to be safer in the future.” | 0.793 (0.406) | 0.896 (0.306) | 0.104 (0.444) | 192 |
|  | 1. “You should go back home and try not to provoke him in the future.” | 0.365 (0.483) | 0.714 (0.453) | 0.346 (0.566) | 191 |
| **ATTITUDES** | | | | |  |
| General attitudes**^ǂǂ^** | ***For each row indicate whether you Strongly Disagree, Disagree, Neither Agree nor Disagree, Agree, or Strongly Agree by circling the corresponding number (5 POINT LIKERT SCALE)*** | | | | |
|  | 1. As a health worker, how I respond to a woman who has suffered violence or sexual abuse is very important. | 7.19 (2.45) | 8.11 (1.91) | 0.924 (2.74) | 192 |
|  | 1. A woman subjected to violence will deny that she has been abused if I ask her about it. | 5.38 (2.72) | 5.26 (2.79) | -0.0916 (3.63) | 191 |
|  | 1. Domestic violence is a private matter and people outside the family should not interfere. | 5.81 (3.23) | 6.42 (3.45) | 0.638 (3.99) | 192 |
|  | 1. It is never a woman’s own fault if she is raped. | 6.52 (2.81) | 7.64 (2.52) | 1.13 (3.35) | 192 |
|  | 1. The woman should have defended herself, she could have avoided being raped. | 3.05 (2.54) | 3.77 (2.9) | 0.729 (3.49) | 192 |
|  | 1. I should not try to convince a woman subjected to domestic violence to leave her violent relationship | 4.82 (2.73) | 4.05 (3.05) | -0.785 (3.72) | 191 |
|  | 1. A woman does not deserve to be abused whether or not she continues to stay with her violent husband. | 5.34 (2.85) | 5.39 (3.15) | 0.0391 (3.61) | 192 |
|  | 1. When interviewing a woman subjected to domestic violence or sexual assault who does not want to talk about details of her story, I should insist. | 3.27 (2.6) | 3.42 (2.75) | 0.156 (3.31) | 192 |
|  | 1. I would feel uncomfortable asking a woman about violence. | 5.74 (2.55) | 6.87 (2.5) | 1.13 (3.26) | 192 |
|  | 1. Sexual abuse only happens to girl children. | 5.61 (3.14) | 6.22 (3.18) | 0.625 (4.09) | 192 |
| Attitudes towards unacceptability of violence**^ǂǂ^** | ***Read each reason below and indicate whether or not you think it is acceptable for a man to hit his wife or girlfriend in that situation? (Yes, it is acceptable \| Sometimes it is acceptable \| No, it is never acceptable; 3 POINT SCALE)*** | | | | |
|  | 1. If she goes out without telling him. | 6.07 (3.9) | 7.44 (3.72) | 1.38 (5.11) | 192 |
|  | 1. If she neglects the children | 5.7 (4.35) | 7.07 (4.16) | 1.37 (5.65) | 193 |
|  | 1. If she argues with him. | 5.94 (3.82) | 6.8 (4.06) | 0.916 (4.94) | 191 |
|  | 1. If she refuses to have sex with him. | 6.47 (3.43) | 7.23 (3.95) | 0.785 (4.65) | 191 |
|  | 1. If she burns the food | 7.67 (2.94) | 8.26 (3.22) | 0.628 (3.88) | 191 |
|  | 1. If he suspects that she is being unfaithful. | 6.04 (3.89) | 7.31 (3.82) | 1.3 (5.06) | 192 |
|  | 1. If he finds out that she is unfaithful. | 4.32 (4.32) | 5.47 (4.42) | 1.17 (5.53) | 192 |
| Perceptions of gender roles**^ǂǂ^** | ***Read each statement below and indicate whether you Strongly Disagree, Disagree, Neither Agree nor Disagree, Agree, or Strongly Agree with the following statement by circling the corresponding number (5 POINT LIKERT SCALE)*** | | | | |
|  | 1. It is the wife's obligation to have sex with her husband whenever he wants it, except when she is sick or menstruating | 5.31 (3.22) | 5.21 (3.35) | -0.147 (3.48) | 187 |
|  | 1. Women and men should share authority in the family | 6.66 (2.92) | 7.71 (2.44) | 1.14 (3.05) | 189 |
|  | 1. A woman's most important role is to take care of her home and cook for her family | 4.93 (3.09) | 5.85 (2.96) | 0.899 (3.43) | 189 |
|  | 1. It is natural (god intended) that men should be the head of the family | 3.78 (3.32) | 4.12 (3.04) | 0.317 (3.84) | 189 |
|  | 1. A wife should obey her husband even if she disagrees | 6.64 (2.58) | 7.28 (2.28) | 0.612 (3.01) | 188 |
|  | 1. A woman should be able to spend her own money according to her own will | 4.02 (2.74) | 4.7 (3.07) | 0.731 (3.4) | 188 |
| Perceptions of professional roles**^ǂǂ^** | ***Read each statement below and indicate whether you Strongly Disagree, Disagree, Neither Agree nor Disagree, Agree, or Strongly Agree with the following statement by circling the corresponding number (5 POINT LIKERT SCALE)*** | | | | |
|  | a. Asking patients about domestic violence is an invasion of their privacy | 7.22 (2.33) | 7.17 (2.66) | -0.0134 (2.86) | 187 |
|  | b. It is humiliating to patients to question them about abuse | 7.24 (2.11) | 7.4 (2.32) | 0.212 (2.74) | 189 |
|  | c.  If I ask non-abused patients about domestic violence, they will get very angry. | 5.44 (2.74) | 5.7 (2.77) | 0.291 (3.22) | 189 |
|  | d. I am afraid of offending the patient if I ask about domestic violence | 6.07 (2.27) | 6.82 (2.32) | 0.722 (3.04) | 187 |
|  | e. Asking about the underlying cause of a patient’s injury does not make a difference to providing quality medical care to the patient | 5.75 (2.65) | 6.54 (2.8) | 0.82 (3.4) | 189 |
|  | f. The way a couple chooses to resolve a conflict is not my business | 5.29 (2.71) | 4.7 (3.12) | -0.608 (4.12) | 189 |
| **PREPAREDNESS** | | | | |  |
| Individual preparedness**^ǂǂ^** | ***How prepared to do you feel for doing the tasks below? (Not at all prepared \| Slightly Prepared \| Somewhat Prepared \| Sufficiently Prepared \| Quite well Prepared; 5 POINT SCALE)*** | | | | |
|  | 1. Identify a woman who has been subjected to violence by signs and symptoms she reports | 5.39 (2.82) | 8.1 (2.76) | 2.72 (3.54) | 190 |
|  | 1. Ask a female patient about whether she has experienced violence from her husband or family | 5.39 (2.92) | 7.69 (2.98) | 2.3 (3.74) | 190 |
|  | 1. Provide care to a woman who is or has been subjected to domestic violence or sexual assault | 5.88 (3.01) | 8.32 (2.62) | 2.47 (3.56) | 190 |
|  | 1. Offer supportive statements to a woman subjected to domestic violence or sexual assault | 6.09 (3.12) | 7.73 (3.16) | 1.6 (3.93) | 189 |
|  | 1. Talk to the woman about her needs and option she may have | 5.32 (2.91) | 7.6 (2.88) | 2.3 (4.01) | 190 |
|  | 1. Document the history and physical examination findings in a patient’s records | 6.68 (3.29) | 8.62 (2.42) | 1.93 (3.74) | 193 |
|  | 1. Assess the immediate level of danger for a woman or child after sexual assault or domestic violence | 5.75 (3.27) | 7.94 (2.9) | 2.19 (3.83) | 193 |
|  | 1. Help the woman create a plan to increase her and her children’s safety | 5.39 (3.26) | 8.25 (2.75) | 2.83 (4.28) | 192 |
|  | 1. Refer the woman to support services available within the community (psychological, legal, shelter, etc.) | 6.23 (3.35) | 8.27 (2.74) | 2.04 (3.96) | 191 |
|  | 1. Understand the Law Against Domestic Violence and my responsibilities as a health provider under the law | 5.41 (3.45) | 8.07 (2.88) | 2.66 (4.34) | 193 |
| Perceived system support**^ǂ^** | ***In providing care to women who experience domestic violence or sexual assault, do you have the following resources and support to help you carry out your tasks? (YES/NO)*** | | | | |
|  | 1. I have a colleague with whom I can get advice on how to respond to a difficult case of domestic violence if I don’t know what to do | 0.791 (0.408) | 0.832 (0.374) | 0.0423 (0.513) | 189 |
|  | 1. I can readily look up information (e.g. either a guide or standard operating procedure) on how to manage cases of domestic violence or sexual assault | 0.675 (0.469) | 0.855 (0.353) | 0.178 (0.531) | 191 |
|  | 1. I have a private space in the facility where I can talk to the woman confidentially about her abuse | 0.733 (0.444) | 0.896 (0.306) | 0.162 (0.501) | 191 |
|  | 1. My supervisor supports me pro-actively asking my patients or clients about whether they are experiencing domestic violence | 0.681 (0.467) | 0.937 (0.243) | 0.258 (0.505) | 190 |
|  | 1. I have names and contact information of people within this facility to whom I can refer the client who discloses violence for additional counselling or psychosocial support | 0.754 (0.432) | 0.891 (0.312) | 0.136 (0.482) | 191 |
|  | 1. I have names and contact information of people outside the facility to whom I can refer the woman for additional psychosocial support | 0.628 (0.485) | 0.917 (0.276) | 0.288 (0.528) | 191 |
| **EMPATHY** | | | | |  |
| Empathy**^ǂǂ^** | ***Read each statement and indicate how frequently you feel or act in the manner described, by circling the corresponding number (Never \| Rarely \| Sometimes \| Often \| Always; 5 POINT SCALE)*** | | | | |
|  | 1. When someone else is feeling excited, I tend to get excited too | 5.62 (2.98) | 5.38 (3.72) | -0.246 (4.35) | 193 |
|  | 1. Other people’s misfortunes do not disturb me a great deal | 6.3 (2.83) | 6.74 (3.29) | 0.456 (3.83) | 192 |
|  | 1. It upsets me to see someone being treated disrespectfully | 5.92 (3.33) | 6.74 (3.5) | 0.846 (4.14) | 192 |
|  | 1. I remain unaffected when someone close to me is happy | 5.96 (2.93) | 5.99 (3.65) | 0.026 (4.65) | 192 |
|  | 1. I enjoy making other people feel better. | 6.92 (3.12) | 7.49 (3.04) | 0.57 (3.56) | 193 |
|  | 1. I have tender, concerned feelings for people less fortunate than me | 5.69 (3.28) | 6.92 (3.22) | 1.2 (4.03) | 191 |
|  | 1. When a friend starts to talk about his\her problems, I try to steer the conversation towards something else | 7.02 (3.37) | 7.93 (3.12) | 0.898 (3.85) | 192 |
|  | 1. I can tell when others are sad even when they do not say anything | 6.22 (2.77) | 7.03 (2.9) | 0.82 (3.6) | 192 |
|  | 1. I find that I am “in tune” with other people’s moods. | 5.38 (2.99) | 6.4 (3.24) | 1.06 (3.83) | 189 |
|  | 1. I do not feel sympathy for people who cause their own serious illnesses | 6.59 (3.29) | 6.53 (3.73) | -0.0798 (4.87) | 188 |
|  | 1. I become irritated when someone cries | 7.03 (3.29) | 7.9 (3.21) | 0.851 (3.77) | 191 |
|  | 1. I am not really interested in how other people feel | 7.49 (3.03) | 8.53 (2.56) | 1.04 (3.16) | 189 |
|  | 1. I get a strong urge to help when I see someone who is upset | 6.7 (3.05) | 7.75 (2.98) | 1.02 (3.64) | 188 |
|  | 1. When I see someone being treated unfairly, I do not feel very much pity for them | 5.55 (3.79) | 6.85 (3.9) | 1.24 (5.2) | 188 |
|  | 1. I find it silly for people to cry out of happiness | 7.4 (3.01) | 7.97 (2.83) | 0.582 (3.77) | 189 |
|  | 1. When I see someone being taken advantage of, I feel kind of protective towards him\her | 6.06 (3.23) | 7.02 (3.11) | 0.961 (3.81) | 190 |

**^ǂ^** *True/false and yes/no questions are presented as proportions (out of 1) and can be interpreted as the proportion of the sample selecting the correct answer for corresponding survey items.*

**^ǂǂ^** *Likert scaled questions were calculated as the proportion of total possible points achieved proportional to 10 (for example, a score of 4/5 on a Likert scale with 5 as the correct answer would be rescaled to a score of 4/5 * 10 = 8) for ease of interpretation and comparison to the main text analyses.*

# Table S3. Survey items used in measurement of each topic area and domain analyzed.

| **Topic Area** | **Survey Item** |
| --- | --- |
| Clinical signs of violence | For each of the following, indicate whether or not it is a **warning sign** that a woman may have been subjected to domestic or sexual violence? |
|  | a.       Repeated unwanted pregnancy? |
|  | b.     She has problems with alcohol or drug abuse? |
|  | c.       Repeated sexually transmitted infections? |
|  | d.      Chronic unexplained pain or inflammation (e.g. pelvic, headaches)? |
|  | e.      Frequent injuries? |
|  | f.        Injuries that do not match the explanation of how they happened? |
|  | g.       Depression, anxiety or chronic stress? |
|  | h.      Thoughts, plans or acts of self-harm or attempted suicide? |
|  | i.        Repeated health consultations with no clear diagnosis? |
| Unacceptability of violence | Read each reason below and indicate whether or not you think it is **acceptable for a man to hit his wife** or girlfriend in that situation? |
|  | a.       If she goes out without telling him. |
|  | b.       If she neglects the children |
|  | c.       If she argues with him. |
|  | d.       If she refuses to have sex with him. |
|  | e.       If she burns the food |
|  | f.        If he suspects that she is being unfaithful. |
|  | g.       If he finds out that she is unfaithful. |
| Professional roles | *Read each statement below and indicate whether you Strongly Disagree, Disagree, Agree, or Strongly Agree with the following statement.* |
|  | a.       Asking patients about domestic violence is an invasion of their privacy |
|  | b.       It is humiliating to patients to question them about abuse |
|  | c.       If I ask non-abused patients about domestic violence, they will get very angry. |
|  | d.       I am afraid of offending the patient if I ask about domestic violence |
|  | e.       Asking about the underlying cause of a patient’s injury does not make a difference to providing quality medical care to the patient |
|  | f.        The way a couple chooses to resolve a conflict is not my business |
| Individual preparedness | **How prepared** to do you feel for doing the tasks below? |
|  | a.       Identify a woman who has been subjected to violence by signs and symptoms she reports |
|  | b.       Ask a female patient about whether she has experienced violence from her husband or family |
|  | c.       Provide care to a woman who is or has been subjected to domestic violence or sexual assault |
|  | d.       Offer supportive statements to a woman subjected to domestic violence or sexual assault |
|  | e.       Talk to the woman about her needs and option she may have |
|  | f.        Document the history and physical examination findings in a patient’s records |
|  | g.       Assess the immediate level of danger for a woman or child after sexual assault or domestic violence |
|  | h.       Help the woman create a plan to increase her and her children’s safety |
|  | i. Refer the woman to support services available within the community (psychological, legal, shelter, etc.) |
|  | j.         Understand the Law Against Domestic Violence and my responsibilities as a health provider under the law |
| Perceived system support | In providing care to women who experience domestic violence or sexual assault, do you have the following resources and support to help you carry out your tasks? |
|  | a.       I have a colleague with whom I can get advice on how to respond to a difficult case of domestic violence if I don’t know what to do |
|  | b.       I can readily look up information (e.g. either a guide or standard operating procedure) on how to manage cases of domestic violence or sexual assault |
|  | c.       I have a private space in the facility where I can talk to the woman confidentially about her abuse |
|  | d.       My supervisor supports me pro-actively asking my patients or clients about whether they are experiencing domestic violence |
|  | e.       I have names and contact information of people within this facility to whom I can refer the client who discloses violence for additional counselling or psychosocial support |
|  | f.        I have names and contact information of people outside the facility to whom I can refer the woman for additional psychosocial support |

# Regression Equation

The probability of improvement in score, $Y_{i},$ was estimated:

$$logit\left( P\left( Y_{i}=1 \right| X_{i} \right))= \beta_{0}+\beta_{1}{female}_{i}+ \beta_{2}{age\_35older}_{i}+ \sum_{j=3}^{5} \beta_{j}I_{P[a]}+\beta_{6}{Liquica}_{i} + \sum_{j=7}^{8} \beta_{j}I_{M[a]}+\beta_{9}{domain}_{i}+ \beta_{10}{previous\_train}_{i}+ \alpha_{p}$$

*Where:*

- ${female}_{i}$ is a binary indicator of participant sex;
- ${age\_35older}_{i}$is a binary indicator of participant age group (dichotomized to <35 and 35+);
- $I_{P[a]}$is a dummy variable indicating the specific position type, $P,$ to which the observation belongs;
- ${Liquica}_{i}$ is a binary indicator of municipality;
- $I_{M[a]}$is a dummy variable indicating the month of training, $M,$ to which the observation belongs;
- ${domain}_{i}$ is a binary indicator of observation sub-domain;
- ${previous\_train}_{i}$is a binary indicator of whether the participant had ever previously participated in violence against women training; and
- $\alpha_{p}$ is a participant identifier random intercept.

Age categories and positions were condensed into broader groups due to small numbers of participants in terminal age categories and positions other than doctor, midwife or nurse (main text, table 2). Month of training was considered as a covariate to account for potential increases in facilitator confidence and competency in delivering trainings as they gained more practice over time. It was not possible to consider years of clinical practice and average weekly patient volume as covariates due to a high degree of missingness in these variables (main text, table 2). For attitudes and perceived preparedness constructs (in which multiple domains were assessed), we included an additional covariate to indicate the sub-domain to which the observation belonged.
